# Diversity and pathogenic characteristics of the *Fusarium* species isolated from minor legumes in Korea

**DOI:** 10.1038/s41598-023-49736-4

**Published:** 2023-12-18

**Authors:** Min Sun Ha, Hyunjoo Ryu, Ho Jong Ju, Hyo-Won Choi

**Affiliations:** 1 Crop Protection Division, National Institute of Agricultural Sciences, Wanju, 55365 Korea; 2 Extension Service Bureau Disaster Management Division, Rural Development Administration, Jeonju, 54875 Korea; 3 Department of Agricultural Biology, Jeonbuk National University, Jeonju, 54896 Korea; 4 Institute of Agricultural Science and Technology, Jeonbuk National University, Jeonju, 54896 Korea

**Keywords:** Microbiology, Plant sciences

## Abstract

Legumes are primarily grown agriculturally for human consumption, livestock forage, silage, and as green manure. However, production has declined primarily due to fungal pathogens. Among them, this study focused on *Fusarium* spp. that cause Fusarium wilt in minor legumes in Korea. Diseased legume plants were collected from 2020 to 2021, and diverse fungal genera were isolated from the internal tissues of the plant roots and stems. *Fusarium* spp. were the most dominant, accounting for 71% of the isolates. They were identified via morphological characteristics and molecular identification. In the pathogenicity test, *Fusarium oxysporum* and *Fusarium fujikuroi* generally exhibited high virulence. The host range investigation revealed that the NC20-738, NC20-739, and NC21-950 isolates infected all nine crops, demonstrating the widest host range. In previous studies, the focus was solely on Fusarium wilt disease in soybeans. Therefore, in this study, we aimed to investigate Fusarium wilt occurred in minor legumes, which are consumed as extensively as soybeans, due to the scarcity of data on the diversity and characteristics of *Fusarium* spp. existing in Korea. The diverse information obtained in this study will serve as a foundation for implementing effective management strategies against *Fusarium*-induced plant diseases.

## Introduction

Legumes are plants belonging to the family Fabaceae whose seeds are sometimes referred to by the term pulses when used as dried grains^1^. Approximately 751 genera and 19,000 species of legumes are known worldwide^2,3^. They are widely distributed and comprise the third largest land plant family following Orchidaceae and Asteraceae, accounting for 7% of flowering plant species^4,5^. Among these, well-known legumes as crops include soybeans (*Glycine max* (L.) Merr), kidney beans (*Phaseolus vulgaris* L.), chickpeas (*Cicer arietinum* L.), lentils (*Lens culinaris* Medik.), mung beans (*Vigna radiate* (L.) Wilczek), adzuki beans (*V. angularis* L.), and clover (*Trifolium repens* L.). Legumes are primarily cultivated for livestock forage and silage, soil-improving green manure, and human consumption, as they are a nutritious food source containing high minerals, carbohydrates, fibers, and protein^2,6^. Accordingly, legumes are grown in numerous countries, such as India, Canada, Myanmar, China, Russia, Türkiye, and Korea, as important protein sources and means of reducing dependency on synthetic pesticides and nitrogen fertilizers^7,8^. According to the crop production statistics published by the National Statistical Office of the Republic of Korea, although domestic legume production has continuously decreased from 172,000 tons in 2013, there has been an increase since 2017, with domestic legume production of 129,925 tons and cultivation area of 63,956 ha in 2021^9^.

In India, the world’s largest producer of legumes, 10%–15% food legume production is lost due to diseases^8^. Various factors cause diseases in legumes, such as parasitic weeds, nematodes, pests, viruses, bacteria, and fungi. Among them, fungi constitute the largest and most important pathogens that affect all plant parts at all growth stages^10^. The major fungal diseases of leguminous crops causing damage worldwide are rust (*Uromyces* spp., *Phakopsora* spp., and *Puccinia* spp.), powdery mildews (*Erysiphe* spp. and *Podosphaera* spp.), downy mildews (*Peronospora* spp.), ascochyta blight (*Ascochyta* spp. and *Phoma* spp.), botrytis gray mold (*Botrytis* spp.), anthracnose (*Colletotrichum* spp.), damping-off (*Pythium* spp., *Rhizoctonia* spp., and *Fusarium* spp.), root rot (*Aphanomyces euteiches*, *Rhizoctonia solani*, *Fusarium* spp., etc.), collar rot (*Sclerotium rolfsii*), Fusarium wilt (*Fusarium* spp.), and white mold (*Sclerotinia* spp.)^11^. In South Korea, 31 fungal diseases have been reported in soybeans, 14 in kidney beans and mung beans, 12 in adzuki beans, and 2 in sword beans. In particular, Fusarium wilt has been studied in some soybeans^12^, but not in minor legumes such as mung bean, kidney bean, adzuki bean, and sword bean. To the best of our knowledge, this is the first study regarding the diversity and pathogenic characteristics of *Fusarium* spp. isolated from minor legumes in Korea.

Link introduced the *Fusarium* genus in 1809^13^; several species in this genus reportedly induce diseases in plants, human, and livestock^14^. Moreover, *Fusarium* genus are well known as taxonomically controversial fungi. Currently, *Fusarium* genus comprises over 300 phylogenetically distinct species distributed among 23 evolutionary lineages referred to as species complexes based on morphological, biological, and phylogenetic species concepts^15^. As plant pathogens, *Fusarium* spp. have caused several significant social impacts in the past, such as the Fusarium wilt caused by *Fusarium oxysporum* f. sp. *cubense*^10^ in the 1960s, which nearly devastated the commercial banana industry. In 2018, the American Phytopathological Society’s List of Plant Diseases reported that more than 81 of the 101 economically important plant species suffered from at least one associated *Fusarium* disease^16^. In leguminous crops, at least 19 species of *Fusarium* have been isolated, and they are primarily responsible for symptoms such as wilt, root rot, sudden death syndrome, and damping-off^17^. Likewise, various *Fusarium* spp. are involved in legume diseases; among them, *F. oxysporum* is considered the major pathogen that causes *Fusarium* wilt^18^. In addition, soybean sudden death syndrome caused by *F. virguliforme*, *F. tucumaniae* and *F. brasiliense* cause serious damage throughout the United States and South America, and in severe cases the disease causes 100% yield loss^19^.

The most suitable strategy to control Fusarium wilt of the crops such as legumes, vegetables, and ornamentals is cultivating resistant varieties^20^. However, it is crucial to have data on the distribution and pathogenic characteristics of domestic *Fusarium* spp. for breeding disease-resistant varieties and diagnosing diseases. Unfortunately, the information for leguminous crops is currently very limited in Korea. Even the available data mostly pertain to soybeans, and there is a lack of studies focused on minor legumes. The aim of the study is to fill this research gap and provide valuable insights into leguminous crop diseases beyond soybeans. To achieve the purpose, this study was conducted as follows; 1) The occurrence of *Fusarium* disease was investigated in minor legumes other than soybeans grown in Korea, 2) *Fusarium* genus isolated from diseased hosts were identified through morphological, cultural, and phylogenetic characteristics, and the species diversity of *Fusarium* distributed in Korea was analyzed, and 3) The differences of virulence toward host plants and their host range of each isolate was investigated.

## Results

### Symptoms and fungal isolation

**Figure 1:**
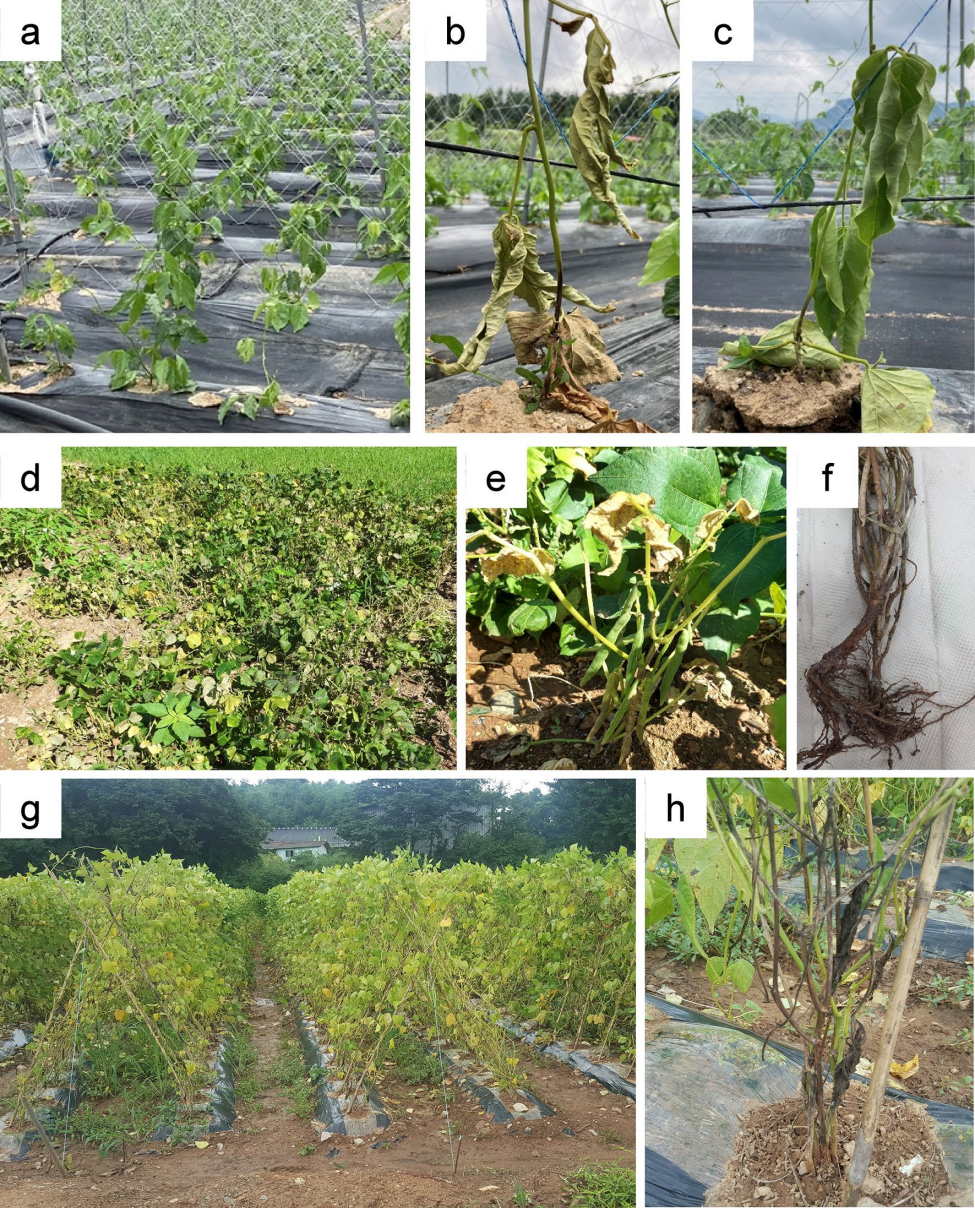
Legumes showing typical wilt symptoms observed in 14 domestic legume plantations in Korea. (**a–c**) Kidney beans in Hongseong, (**d–f**) Adzuki beans in Yeosu, and (**g–h**) Sword beans in Hwasun.

Wilt symptoms were observed in 14 legume cultivation fields in Chungnam, Chungbuk, Gyeongnam, and Jeonnam province during 2020–2021 in Korea. However, there was a difference in the severity of the wilt depending on the cultivation fields. The results revealed that the incidence of wilt symptoms in all the legume fields was 1%–5%, mostly occurring during the high-temperature period of June–September, following the middle growth period of the legume plants. The observed wilt symptoms of the legume plants included yellowing of leaves, browning of stems and roots, root rot, stunting, wilting, and plant death (Fig. 1). As a result of isolating fungi from diseased plant samples, a total of 41 fungi were obtained. Among them, *Fusarium* spp. were isolated the most with 29 followed by *Colletotrichum* spp. were isolated with 4, *Macrophomina* spp. with 3, *Rhizoctonia* spp. with 2, and *Phytophthora* sp., *Pythium* sp. and *Lasiodiplodia* sp. with 1 each (Supplementary Fig. S1). A total of 6 *Fusarium* isolates were obtained from diseased kidney bean samples collected from 4 fields, and 9 isolates of mung bean were isolated from 5 fields. Eight *Fusarium* isolates were obtained from 3 adzuki bean fields, and 6 isolates were isolated from 2 sword bean fields. Each isolate was obtained from a different diseased plant sample.

### Morphological and cultural characteristics

Following the investigation of the morphological characteristics of macroconidia, microconidia, chlamydospores, sporodochia, and aerial mycelia, 29 isolates of *Fusarium* were classified into 4 species complexes: *F. oxysporum* species complex (FOSC), *F. solani* species complex (FSSC), *F. fujikuroi* species complex (FFSC), and *F. incarnatum-equiseti* species complex (FIESC). Six isolates obtained from kidney beans were classified as FSSC (3 isolates), FOSC (2 isolates), and FFSC (1 isolate). Nine isolates of mung beans were classified as FSSC (4 isolates), FOSC (3 isolates), and FIESC (2 isolates). Eight isolates of adzuki beans were classified as FSSC (3 isolates), FOSC (2 isolates), and FFSC (3 isolates). Lastly, six isolates of sword beans were classified as FSSC (3 isolates) and FFSC (3 isolates).

**Figure 2:**
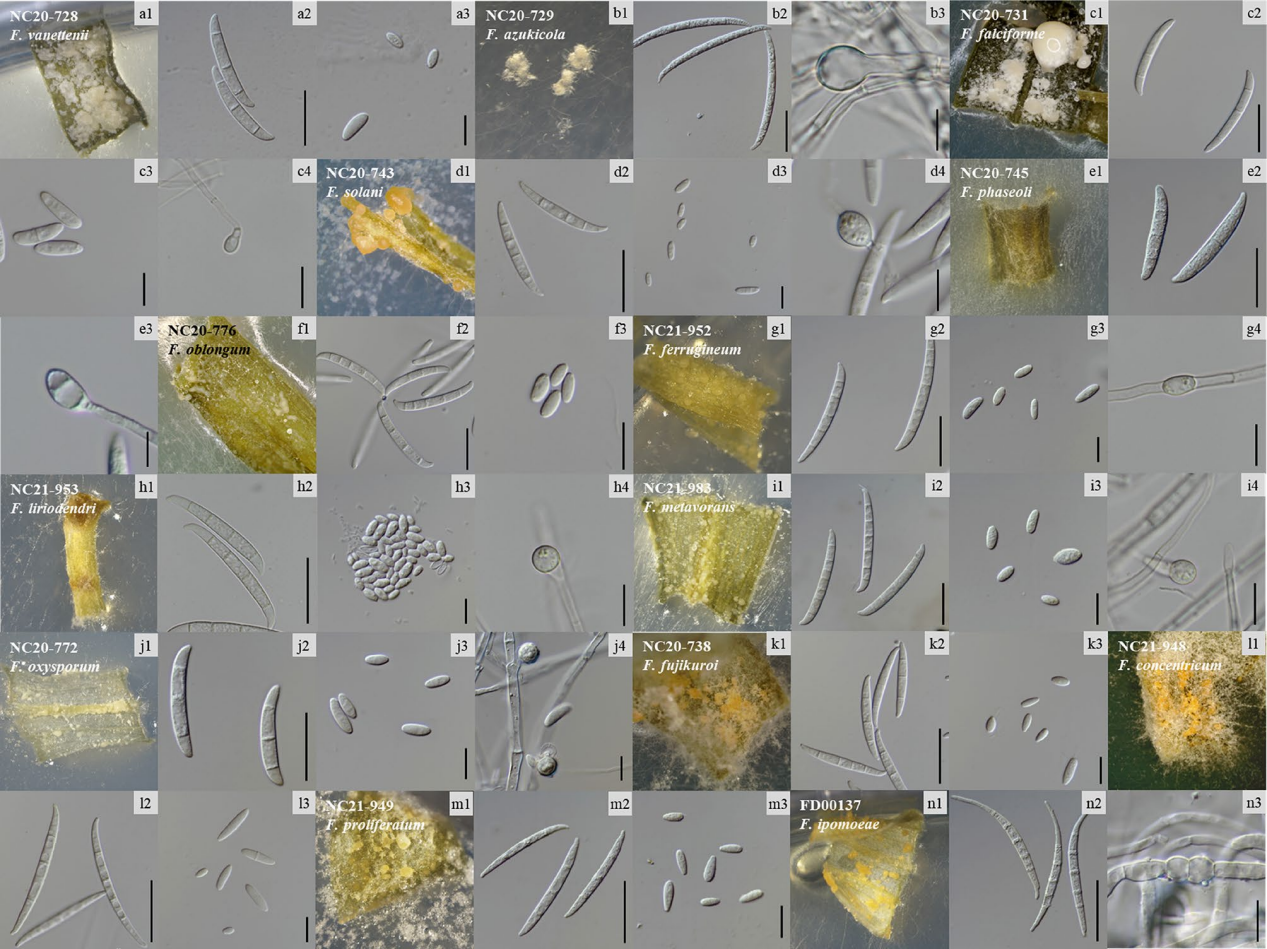
Morphological microscopic images of 14 *Fusarium* species. (**a1, b1, c1, d1, e1, f1, g1, h1, i1, j1, k1, l1, m1, n1**) Sporodochia. (**a2, b2, c2, d2, e2, f2, g2, h2, i2, j2, k2, l2, m2, n2**) Macroconidia, Scale bar = 25 µm. (**a3, c3, d3, f3, g3, h3, i3, j3, k3, l3, m3**) Microconidia, Scale bar = 10 µm. (**b3, c4, d4, e3, h4, i4, j4, n3**) Chlamydospore, Scale bar = 10 µm. (**a–i**) *Fusarium solani* species complex (FSSC), (**j**) *F. oxysporum* species complex (FOSC), (**k–l**) *F. fujikuroi* species complex (FFSC), and (**n**) *F. incarnatum-equiseti* species complex (FIESC).

Among the 29 *Fusarium* isolates, 13 (45%) were identified as FSSC. The macroconidia of these isolates were plump and usually straight with three to five septa, they had oval or obovoid microconidia. The sporodochia of FSSC formed on carnation leaf agar (CLA) were white to beige and usually formed chlamydospores. However, the sporodochia of the NC20-729 and NC20-745 isolates rarely formed, and their microconidia were not observed. Among them, the macroconidia of the NC20-729 isolate were considerably larger and thinner than those of other isolates belonging to FSSC. In addition, the color of sporodochia in the NC20-743 isolate was pale orange rather than white to beige, which differed from the general characteristics of FSSC. Moreover NC20-728 and NC20-776 isolates didn’t form chlamydospores. The detailed cultural and morphological characteristics of isolates belonging to FSSC are described in Supplementary Table S1. Seven isolates (24%) were identified as FOSC. They had slightly curved macroconidia with three to four septa and oval or clavate microconidia. In this species complex, sporodochia were generally absent, and when present, they were orange. However, the NC20-772 isolate specifically formed white to beige–colored sporodochia. The detailed cultural and morphological characteristics of the FOSC isolates are described in Supplementary Table S2. In addition, seven isolates (24%) were identified as FFSC, which formed slender macroconidia with no significant curvature. The microconidia were formed in chains and did not form chlamydospore. The detailed cultural and morphological characteristics of the FFSC isolates are described in Supplementary Table S3. Only the NC21-948 isolate formed short chains of microconidia, whereas all the other FFSC isolates formed long chains of microconidia (data not shown). Finally, two isolates (7%) were identified as FIESC, which formed elongated and whip-like macroconidia but did not form microconidia. The sporodochia observed on CLA were orange to beige in color. The detailed cultural and morphological characteristics of the FIESC isolates are described in Supplementary Table S4. Morphological characteristics images of representative isolates of 14 *Fusarium* species are shown in Fig. 2.

The cultural characteristics on PDA media of *Fusarium* isolate tended to be similar within the same species. However, some species exhibited different growth rates, phenotypes, and pigmentation owing to their intraspecies diversity. For example, unlike most *Fusarium* spp., NC20-729 of *F. azukicola* and NC20-745 of *F. phaseoli* exhibited a slow growth rate. The previously described morphological and cultural characteristics of 14 *Fusarium* spp. comprising 29 isolates are described in Supplementary Table S5.

### Molecular identification by phylogenetic analysis

**Figure 3:**
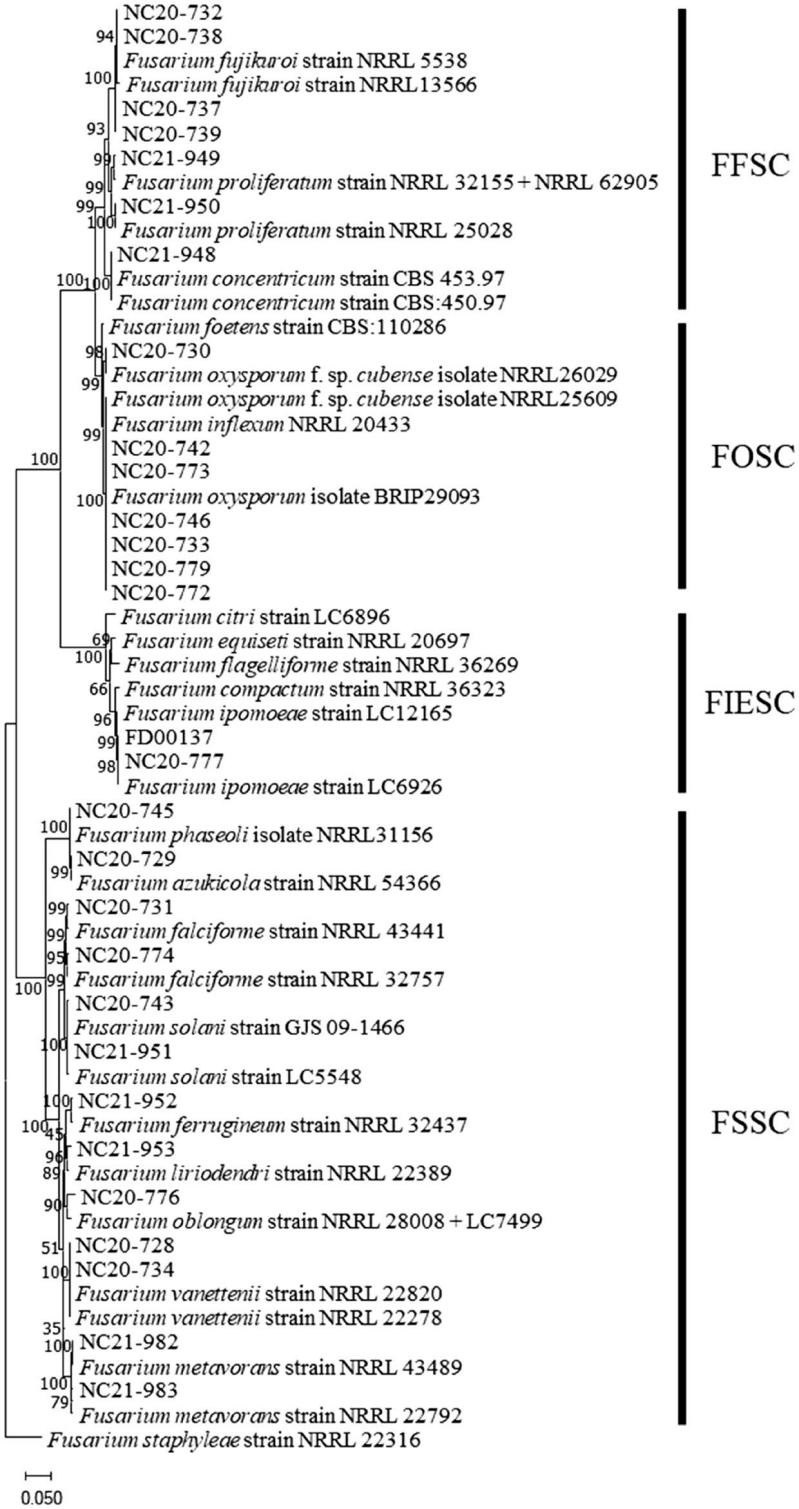
Phylogenetic trees of *Fusarium* species obtained from wilted legume plants in Korea. The trees were generated using Maximum likelihood analysis of translation elongation factor 1α (TEF) and RNA polymerase II second largest subunit (RPB2) genes nucleotide sequences. The number in each branch indicates bootstrap values obtained after a bootstrap test with 1,000 replications. The scale bar represents 0.05 nucleotide substitution per site.

For accurately identifying the 29 *Fusarium* isolates to the species level, the nucleotide sequences of the translation elongation factor 1 alpha (TEF) and RNA polymerase II second largest subunit (RPB2) regions were analyzed, and their amplification sizes were 600–800 bp and 1,800–2,000 bp, respectively (Supplementary Fig. S2). The phylogenetic tree for the 29 isolates was divided into 4 species complex; FSSC, FOSC, FFSC, FIESC (Fig. 3). The FSSC isolates included *F. vanettenii*, *F. azukicola*, *F. falciforme*, *F. solani*, *F. phaseoli*, *F. oblongum*, *F. ferrugineum*, *F. liriodendri*, and *F. metavorans*. The FFSC isolates included *F. fujikuroi*, *F. concentricum*, and *F. proliferatum*. However, all seven FOSC isolates included *F. oxysporum*, and two FIESC isolates included *F. ipomoeae*. The full list of these isolates with their hosts and accession numbers are provided in Supplementary Table S6. Our results reveal that 14 *Fusarium* spp. were recovered from the minor legumes exhibiting typical wilt symptoms, among which *F. oxysporum* was the most common species (seven isolates) followed by *F. fujikuroi* (four isolates).

### Pathogenicity test

**Figure 4:**
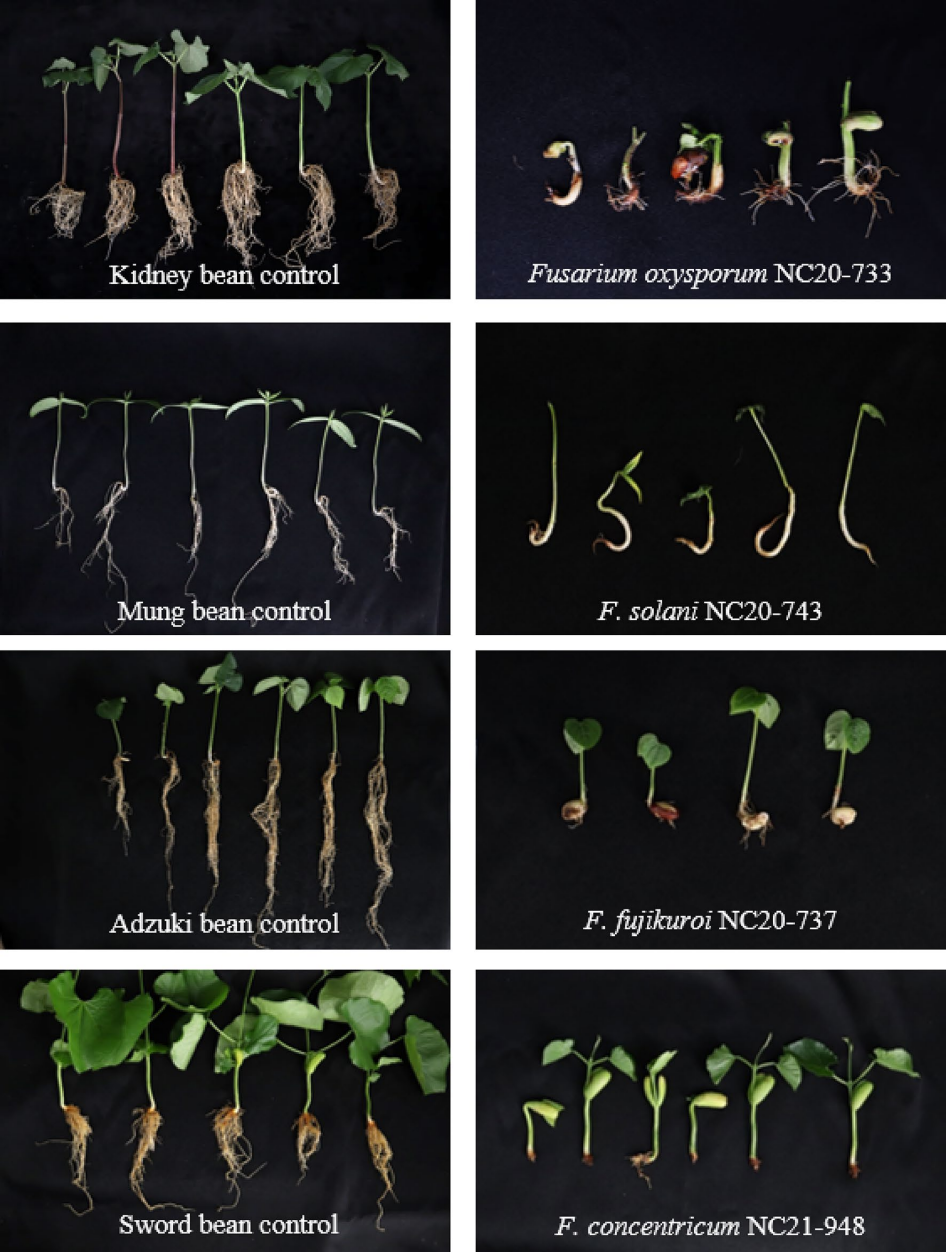
Pathogenicity test for the original host plant. Control plants (left) and diseased plants inoculated with *Fusarium* isolates (right).

As a result of performing a pathogenicity test on 29 *Fusarium* isolates on the isolated host plants, each isolate showed different virulence (Table 1). Even isolates identified as the same species, such as *F. poliferatum* isolated from sword bean, showed different pathogenic responses. The isolates evaluated as highly virulent had an average disease index of 3 or greater, including five isolates of *F. oxysporum*, four isolates of *F. fujikuroi*, and a single isolate for each of *F. solani*, *F. azukicola*, *F. vanettenii*, *F. proliferatum* and *F. concentricum*. Conversely, the *F. falciforme*, *F. metavorans*, and *F. ipomoeae* isolates were less virulent or nonpathogenic. Isolates obtained from kidney beans generally showed high virulence and were also found to cause wilt disease for the first time in Korea, except for *F. phaseoli*. The highly virulent isolates identified in the study displayed aggressive pathogenicity, leading to root rot and xylem blockage in their host plants (Fig. 4). Because of these pathogenic effects, water absorption in the host plants was obstructed, resulting in the inhibition of their growth. Moreover, only the first leaf was grown in the aboveground parts of the plants. The pathogenicity test on the 29 obtained isolates revealed that each isolate exhibited different pathogenicity even when they belonged to the same species.

**Table 1:** Pathogenicity of the 29 *Fusarium* isolates obtained from wilted minor legumes against their original hosts.

Original host	Isolate^a^	Species^b^	Mean of disease index^c^ (standard deviation)			
			Kidney bean	Mung bean	Adzuki bean	Sword bean
Kidney bean	NC20-728	F. vanettenii*	2.8 (± 0.37)			
	NC20-732	F. fujikuroi*	4 (± 0.00)			
	NC20-733	F. oxysporum*	4 (± 0.00)			
	NC20-734	F. vanettenii*	3 (± 0.00)			
	NC20-745	*F. phaseoli*	2.7 (± 0.47)			
	NC20-746	F. oxysporum*	3.8 (± 0.37)			
Mung bean	NC20-729	F. azukicola*		4 (± 0.00)		
	NC20-730	*F. oxysporum*		3.2 (± 0.37)		
	NC20-731	*F. falciforme*		0.3 (± 0.47)		
	NC20-742	*F. oxysporum*		2.2 (± 0.69)		
	NC20-743	*F. solani*		4 (± 0.00)		
	NC20-776	F. oblongum*		1.8 (± 0.37)		
	NC20-777	*F. ipomoeae*		1.3 (± 0.47)		
	NC20-779	*F. oxysporum*		3.8 (± 0.43)		
	FD00137	*F. falciforme*		0.7 (± 0.94)		
Adzuki bean	NC20-737	F. fujikuroi*			4 (± 0.00)	
	NC20-738	F. fujikuroi*			4 (± 0.00)	
	NC20-739	F. fujikuroi*			3 (± 0.63)	
	NC20-772	F. oxysporum*			1.5 (± 0.50)	
	NC20-773	F. oxysporum*			3.2 (± 0.69)	
	NC20-774	*F. falciforme*			0.8 (± 0.75)	
	NC21-982	*F. metavorans*			1.2 (± 0.75)	
	NC21-983	*F. metavorans*			0.8 (± 0.40)	
Sword bean	NC21-948	F. concentricum*				4 (± 0.00)
	NC21-949	*F. proliferatum*				0 (± 0.00)
	NC21-950	F. proliferatum*				3 (± 0.00)
	NC21-951	*F. solani*				1 (± 0.00)
	NC21-952	*F. ferrugineum*				1 (± 0.00)
	NC21-953	*F. liriodendri*				2.7 (± 1.11)
	CONTROL		0 (± 0.00)	0 (± 0.00)	0 (± 0.00)	0 (± 0.00)

^a^Disease index of NC20-737, NC20-738, and NC20-739 were cited from Ha et al.^43^.

^b^ *New pathogens that have not been reported in Korea so far, in each host.

^c^Disease index 0 = no symptoms, 1 = root necrosis and root loss < 30%, 2 = root necrosis and root loss 31–60%, 3 = root necrosis, root loss > 61%, and poor growth, and 4 = complete necrosis of root tissue and no roots or plants death.

### Host range investigation

Nineteen *Fusarium* isolates were selected to investigate the host range based on the results of the pathogenic characteristics of all the *Fusarium* spp. (Table 2). Investigating the host range of nine kinds of crops belonging to the leguminous and gramineous plants using these selected isolates revealed a very diverse host range for each isolate (Table 3). Duncan's Multiple Range Test (DMRT) using R program (Lucent Technologies, USA) revealed the difference in the incidence of the 19 isolates at a 5% significance level. The FSSC isolates did not cause wilt disease in rice and maize as gramineous hosts even if these isolates were highly virulent. However, in FOSC, the NC20-730 and NC20-773 isolates caused wilt disease in rice as well as legumes (Supplementary Fig. S3). Unlike other species complexes, all the FFSC isolates caused wilt disease in rice and generally had a wide host range. Specifically, the NC20-738 and NC20-739 isolates of *F. fujikuroi* and the NC21-950 isolate of *F. proliferatum* demonstrated significant pathogenicity in all the nine plants. Conversely, the NC20-772 isolate, which exhibited low virulence in the pathogenicity assay, did not cause wilt disease in all the tested plants except for adzuki beans.

**Table 2:** List of the pathogenic *Fusarium* isolates that were selected for host range investigation.

Species complex^a^	Species	Isolate	Region	Pathogenicity^b^
FSSC	*F. vanettenii*	NC20-728	Boryeong, Chungnam	++
	*F. azukicola*	NC20-729	Yeosu, Jeonnam	+++
	*F. vanettenii*	NC20-734	Boryeong, Chungnam	++
	*F. solani*	NC20-743	Hongseong, Chungnam	+++
	*F. phaseoli*	NC20-745	Hongseong, Chungnam	++
	*F. oblongum*	NC20-776	Miryang, Gyeongnam	+
	*F. liriodendri*	NC21-953	Hwasun, Jeonnam	++
FOSC	*F. oxysporum*	NC20-730	Yeosu, Jeonnam	+++
	*F. oxysporum*	NC20-733	Boryeong, Chungnam	+++
	*F. oxysporum*	NC20-746	Hongseong, Chungnam	+++
	*F. oxysporum*	NC20-772	Miryang, Gyeongnam	+
	*F. oxysporum*	NC20-773	Miryang, Gyeongnam	+++
	*F. oxysporum*	NC20-779	Miryang, Gyeongnam	+++
FFSC	*F. fujikuroi*	NC20-732	Seocheon, Chungnam	+++
	*F. fujikuroi*	NC20-737	Yeosu, Jeonnam	+++
	*F. fujikuroi*	NC20-738	Yeosu, Jeonnam	+++
	*F. fujikuroi*	NC20-739	Yeosu, Jeonnam	++
	*F. concentricum*	NC21-948	Hwasun, Jeonnam	+++
	*F. proliferatum*	NC21-950	Hwasun, Jeonnam	++

^a^ FSSC, *F. solani* species complex; FOSC, *F. oxysporum* species complex; FFSC, *F. fujikuroi* species complex; FIESC, *F. incarnatum-equiseti* species complex.

^b^+: disease index 1–2, ++: disease index 2.1–3, +++: disease index 3.1–4.

**Table 3:** Host range of 19 selected *Fusarium* spp. isolates that were collected from the wilted minor legumes.

Species complex^a^	Isolate	Origin	Mean of disease index^b^ or occurrence of disease^c^ on hosts															
			Kidney bean		Sword bean		Mung bean		Adzuki bean		Cowpea		Soybean		Lentil bean		Rice	Corn
FSSC	NC20-728	Kidney	2	de^d^	1	d	1	f	2	de	1.5	e	0.9	gh	1	g	−	−
	NC20-729	Mung	4	a	2	c	4	a	4	a	4	a	3.7	ab	2.5	de	−	−
	NC20-734	Kidney	2	de	2	c	1	f	0.6	fg	2.5	d	1.8	ef	1	g	−	−
	NC20-743	Mung	1.7	e	4	a	2	e	0.4	fg	1	ef	3	bcd	0.8	g	−	−
	NC20-745	Kidney	4	a	1	d	3.2	bc	3	b	4	a	0.6	h	4	a	−	−
	NC20-776	Mung	3	b	2	c	1.8	e	1	f	2.5	d	3	bcd	1.7	f	−	−
	NC21-953	Sword	1.6	e	3.6	a	0.8	fg	4	a	1.4	e	2.7	cd	2	ef	−	−
FOSC	NC20-730	Mung	3	b	2.4	bc	4	a	2.8	bc	2.8	cd	4	a	3	cd	+	−
	NC20-733	Kidney	3	b	2.3	bc	3	cd	4	a	3.6	ab	3.3	abc	3.8	ab	−	−
	NC20-746	Kidney	2.7	bc	1.2	d	2.4	de	2	de	2.6	d	2.4	de	2.5	de	−	−
	NC20-772	Adzuki	0	g	0.2	e	0.2	gh	1	f	0	g	1	gh	0.1	h	−	−
	NC20-773	Adzuki	4	a	3.5	a	3.8	ab	3	b	2.7	cd	3.4	abc	2.1	ef	+	−
	NC20-779	Mung	1	f	1	d	2.1	e	0.8	fg	0.4	fg	1.4	fg	3	cd	−	−
FFSC	NC20-732	Kidney	2.4	cd	3.4	a	0.2	gh	1.8	e	0.6	fg	2.3	de	2.7	d	+	+
	NC20-737	Adzuki	3.2	b	2.8	b	3	cd	2.2	cde	3.4	abc	3	bcd	2.7	d	+	−
	NC20-738	Adzuki	4	a	4	a	4	a	4	a	4	a	4	a	4	a	+	+
	NC20-739	Adzuki	4	a	3.8	a	4	a	2.6	bcd	3	bcd	4	a	3	cd	+	+
	NC21-948	Sword	4	a	4	a	4	a	4	a	4	a	4	a	4	a	+	−
	NC21-950	Sword	4	a	4	a	4	a	4	a	3.9	a	2.8	cd	3.4	bc	+	+
Control			0	g	0	e	0	h	0	g	0	g	0	h	0	h	−	−

^a^FSSC, *F. solani* species complex; FOSC, *F. oxysporum* species complex; FFSC, *F. fujikuroi* species complex; FIESC, *F. incarnatum-equiseti* species complex.

^b^Disease index 0 = no symptoms, 1 = root necrosis and root loss < 30%, 2 = root necrosis and root loss 31%–60%, 3 = root necrosis, root loss > 61%, and poor growth, 4 = complete necrosis of root tissue and no roots or plants death.

^c^ +, Occurrence of disease; −, nonoccurrence of disease.

^d^Duncan’s Multiple Range Test (DMRT) for mean comparison of disease index within each host (*P* < 0.05).

## Discussion

This study was undertaken due to the limited research about Fusarium wilt conducted on leguminous crops other than soybeans, which has resulted in insufficient data in Korea. Up-to-date information on the distribution and characteristics of pathogens is required for the development of resistant varieties, disease diagnosis, and effective disease control measures. In this study, several genera including *Fusarium* spp., *Colletotrichum* spp., *Macrophomina* spp., *Rhizoctonia* spp., *Pythium* sp., *Phytophthora* sp., and *Lasiodiplodia* sp. were isolated from wilted legumes as a result of plant sampling and fungal isolation. The results indicate that among the various fungal genera isolated from the wilted legumes, *Fusarium* spp. were the most dominant, accounting for 71% of the recovered isolates (Supplementary Fig. S1). This finding emphasizes the significant role of *Fusarium* species as the major causal pathogen of wilt symptoms in legumes in the studied area. The previous study conducted in Korea from 2014 to 2016 reported similar results, with *Fusarium* spp. being the most frequently isolated genus, accounting for 79% of the isolates from soybeans^12^. This consistency across different studies in Korea suggests the persistence and prevalence of *Fusarium* spp. as important pathogens affecting legumes. On the other hand, the Chinese study related to soybeans revealed different results, with different fungal genera being isolated, including *Fusarium* spp., *Alternaria* sp.*, Aspergillus* sp.*, Botryosphaeria* sp.*, Colletotrichum* sp.*, Corynespora* sp.*,* and *Diaporthe* sp.^21^. The variation in the distribution of fungal pathogens and dominant species between Korea and China may be influenced by factors such as regional variations, environmental conditions, and different cultivation practices. Therefore, future research is needed to investigate the distribution and density of pathogens by collecting samples by climate region and growth period. This will provide a more comprehensive understanding of the pathogen dynamics and their impact on legume crops.

The 29 *Fusarium* isolates of this study were classified into 4 species complexes (FOSC, FSSC, FFSC, and FIESC) according to morphological and cultural characteristics (Fig. 2), and 14 species were identified through TEF (translation elongation factor 1 alpha) and RPB2 (RNA polymerase II second largest subunit) gene sequencing (Fig. 3). However, the NC20-745 isolate could not be clearly distinguished from *F. phaseoli* and *F. crassistipitatum* due to the lack of differences in the nucleotide sequence used for molecular identification. According to Aoki et al.^22^, *F. crassistipitatum* can be distinguished from *F. phaseoli* by forming yellow colonies on potato dextrose agar (PDA) medium. Based on the fact that the NC20-745 isolate forms white colonies, it has been identified as *F. phaseoli* (Supplementary Table S1). Among the 14 species, *F. oxysporum* was the most common with 7 isolates (24%), followed by 4 isolates of *F. fujikuroi*, 2 isolates of *F. solani, F. vanettenii*, *F. falciforme, F. metavorans, F. proliferatum, F. ipomoeae*, and 1 isolate of *F. azukicola, F. phaseoli, F. oblongum, F. ferrugineum, F. liriodendri, F. concentricum.* In a previous study conducted in Korea, the frequency of 53 *Fusarium* strains isolated from soybeans was as follows: *F. solani* (43%), *F. oxysporum* (34%), *F. asiaticum* (9%), *F. fujikuroi* (8%), and *F. commune* (6%)^12^. In the UK, the isolate frequency of 33 *Fusarium* strains isolated from leguminous crops was as high as 30% for *F. coeruleum,* followed by *F. redolens* (18%), *F. avenaceum* (15%), *F. oxysporum* (9%)*, F. sambucinum* (9%), *F. graminearum* (6%)*, Fusarium* spp. (6%), *F. solani* (3%)*,* and *F. equiseti* (3%)^23^. In Spain, *F. oxysporum, F. solani*, and *Fusarium* spp. were isolated from chickpea with wilting and root rot symptoms^24^. Among them, *F. oxysporum* was mainly isolated from dead or dying plants and was the only fungus isolated from plants showing early wilting symptoms. From this point of view, it is thought that the reason why various *Fusarium* spp. could be isolated in this study was because the investigation was conducted in the late stage of growth when the wilting symptoms were evident. As such, *Fusarium* spp. involved in legume wilt and root rot are very diverse, future research needs to investigate the diversity and isolation frequency of *Fusarium* spp. through sample collection according to the growth period of domestic legumes and continuously monitor pathogens.

However, because not all of these isolated *Fusarium* spp. are pathogenic, pathogenicity tests were performed on the original hosts from which each strain was isolated. The results revealed that four isolates of *F. fujikuroi* (100%), five of *F. oxysorum* (71%), one of *F. solani*, *F. proliferatum*, and *F. ipomoeae* (50%), two of *F. vanettenii* (100%), and one of *F. phaseoli*, *F. concentricum*, *F. oblongum*, and *F. azukicola* (100%) had a disease severity of 2.5 or higher (Table 1). Through this, many additional pathogens that were not reported in List of Plant Diseases in Korea were newly identified^25^. These include *F. azukicola* and *F. oblongum* for mung bean, *F. fujikuroi*, *F. oxysporum*, and *F. vanettenii* for kidney bean, *F. fujikuroi* and *F. oxysporum* for adzuki bean, and *F. concentricum* and *F. proliferatum* for sword bean. In particular, in the case of *F. azukicola*, this is the first report in Korea. However, when it was first reported as a new species in Japan^26^, it was isolated from red beans, but there is a difference in that it was isolated from mung beans in Korea. The major pathogen of Fusarium wilt is known to be *F. oxysporum*, but similar to the results of this study, various other *Fusarium* spp. have also been frequently reported as pathogens of wilt and root rot in previous studies^12,17,26,27^. Therefore, research on the diversity of other *Fusarium* species should continue to be conducted because they may become problematic pathogens like *F. oxysporum.* Whereas three isolates of *F. falciforme* (100%), two of *F. metavorans* (100%), one of *F. proliferatum* (50%), and one of *F. ferrugineum* (100%) were nonpathogenic. As such, not all of the 14 isolated *Fusarium* spp. are pathogenic, and even the same species showed different pathogenicity depending on the isolate. Arias et al.^17^ reported that only one strain of 14 *F. oxysporum* strains from infected soybeans caused root rot disease. Likewise, other *Fusarium* spp. showed significant differences in pathogenicity according to strains, which was consistent with the results of this study. In addition, the severity of disease by *Fusarium* spp. was also different between studies. In the US, *F. graminearum* is the most pathogenic followed by *F. virguliforme*, *F. proliferatum*, *F. sporotrichioides*, and *F. solani*^17^. However, in China, *F. proliferatum* is reportedly the most pathogenic followed by *F. fujikuroi*, *F. sulawense*, and *F. luffae*.^21^ These results are attributed to complex differences between countries, including dominant species, cultivated legume species, cultivation environments, and cropping systems. Therefore, it is thought that in-depth investigation according to region, cultivation environment, and cropping system should be conducted in Korea.

As a result of investigating the host range of the 19 selected isolates on 7 leguminous plants and 2 gramineous plants, it was observed that most isolates showed polyxenic (Table 2, 3). Most *Fusarium* isolates did not cause wilt disease in corn; however, three isolates, namely NC20-738, NC21-739 of *F. fujikuroi*, and NC21-950 of *F. proliferatum*, were found to induce wilt disease in all nine crops, including corn. These three isolates were identified as having the widest host range, as they exhibited the ability to infect and cause disease in multiple plant species. Similarly, a previous study conducted by Amatulli et al.^28^ reported that *F. fujikuroi* and *F. proliferatum* have a broad host range, encompassing various plant species, such as corn, asparagus, fig, onion, palm, pine, and rice. Considering the findings of both the previous and the current study, it can be concluded that *F. fujikuroi* and *F. proliferatum* have at least 14 known host species. This indicates their versatility and ability to infect a diverse range of plants, underscoring their significance as potential pathogens with significant agricultural implications.

Furthermore, all isolates belonging to FFSC were found to cause wilt disease in rice. In particular, since *F. fujikuroi* is known to be the causal pathogen of bakanae disease in rice, the immersion inoculation method was additionally performed to verify whether the *F. fujikuroi* strain isolated from legumes could cause bakanae disease^29^. The results revealed that all four *F. fujikuroi* isolates increased the height of rice and caused bakanae disease (data not shown). This finding aligns with the results of a previous study conducted by Choi et al.^12^, which confirmed that *F. fujikuroi* isolated from soybeans could induce bakanae disease in rice. Conversely, when *F. fujikuroi* isolated from rice was inoculated into soybeans (cv. Daewon, Poongwon, Taegwang, Wooram), the stems were abnormally elongated and eventually the plants died with symptoms similar to bakanae disease in rice (data not shown). Thus, it was found that *F. fujikuroi* can cross-infect rice and legumes with each other. Additional research is crucial to address potential problems that may arise in Korea due to the cropping system involving rice paddy rotation and double cropping with legumes. Three isolates of *F. oxysporum* and one isolate of *F. azukicola* also caused wilt disease in all seven legumes. With respect to *F. azukicola*, Aoki et al.^26^ reported that eight strains of *F. azukicola* isolated from Japan also caused root rot in adzuki beans, kidney beans, mung beans, and soybeans. Thus, it is likely to become a problematic fungal pathogen in the near future. As such new pathogens may exist in the future, continuous pathogen identification and host range monitoring are highly recommended.

Currently, research regarding the Fusarium wilt of legumes in Korea is insufficient; hence, information regarding the existing *Fusarium* spp. pathogens is lacking. Therefore, by investigating previously unreported Fusarium wilt pathogens and their pathogenic characteristics and host range, this study fills a critical knowledge gap in understanding the diversity and pathogenic properties of legume pathogens. The findings of this study can be used for future research in effective Fusarium wilt management strategies, including breeding of wilt-resistant varieties and cultivation control methods such as crop rotation.

## Methods

### Sample collection and isolation of the fungi

Experimental research and field studies on plants, including the collection of plant material, complied with relevant institutional, national, and international guidelines and legislation. And we have permission to collect legumes. From 2020 to 2021, 53 samples exhibiting wilt symptoms from minor legumes such as kidney beans, adzuki beans, mung beans, sword beans were collected from 14 domestic legume plantations, in Hongseong, Boryeong, Seocheon, Yeosu, etc. (Supplementary Fig. S4). To isolate the fungi from the samples, the discolored internal tissues of the root and stem were cut into small pieces (5 × 5 mm). The surface-sterilized sample pieces were placed on water agar (WA) and incubated at 25 ℃ in the dark. After 3–5 days of incubation, single spore was isolated by the single spore isolation method^30^. Then, only pure fungal cultures were transferred to PDA slants and stored at 10 ℃ till further use in the following assays.

### Morphological identification and characterization of fungal isolates

The isolates were cultured on CLA media^31,32^ at 25 ℃ for 14 days under near ultraviolet (NUV)/dark (12 h/12 h) incubation conditions to investigate the morphological characteristics of the fungal isolates. Following incubation, the morphological characteristics, such as the shape and size of microconidia, macroconidia, presence or absence and color of sporodochia were investigated^33^. To investigate the cultural characteristics, the isolates were inoculated on PDA and cultured at 25 ℃ in the dark for 7 days. Following incubation, the cultural characteristics, including colony growth rate, aerial mycelial color and texture, and colony pigmentation, were investigated^33^.

### DNA extraction

Genomic DNA was extracted from the mycelial powder using Maxwell® RSC PureFood GMO and Authentication Kit (Promega, Madison, WI, USA) according to the manufacturer’s instructions. Each fungal isolate was individually inoculated by placing three to five pieces of PDA with mycelia into 20 ml potato dextrose broth (Difco, Bergen, USA) and then incubated at 25 ℃ for 5–7 days. Following incubation, the growing fungal mycelia were filtrated using a sterilized piece of miracloth. The harvested mycelia were completely dried via freeze-drying overnight, and then ground using sterilized beads and a homogenizer to prepare mycelial powder. The mycelial powder was vortexed with 20 µL RNase A and 30 µL proteinase K and then incubated in a heating block at 65 ℃ for 30 min. After incubation, they were centrifuged at 14,000 rpm for 5 min, and 400 µL supernatant was recovered. The fungal genomic DNA was extracted from this supernatant using Maxwell® RSC Kit and stored at − 20 ℃ till further use in the subsequent assays.

### Polymerase chain reaction amplification

For the molecular identification of the *Fusarium* isolates, the TEF and RPB2 coding regions were selected^22^. TEF was amplified using EF1 and EF2 primers, and RPB2 was amplified using 5f2 and 11aR primers^34^ (Supplementary Table S7). Polymerase chain reaction (PCR) amplification was performed using 50 µL mixture, containing 3 µL DNA templates (33 ng/µL), 3 µL each F/R primer (5 pmol/µL), 5 µL 10X *n*Taq-Tenuto buffer (Mg^2+^ plus), 5 µL 2 mM dNTP mixture, 30 µL sterile water, and 0.5 µL *n*Taq-Tenuto polymerase. The PCR conditions for DNA amplification of the TEF region included initial denaturation at 95 ℃ for 4 min; 34 cycles of denaturation at 95 ℃ for 30 s, annealing at 58 ℃ for 45 s, extension at 72 ℃ for 50 s; and a final extension at 72 ℃ for 7 min^35^. For the RPB2 region, the PCR conditions were 95 ℃ for 2 min; 34 cycles of denaturation at 95 ℃ for 30 s, annealing at 56 ℃ for 30 s, extension at 72 ℃ for 2 min; and a final extension at 72 ℃ for 5 min^36^.

### DNA purification and sequence analysis

The final PCR products were observed via electrophoresis on a 1.4% agarose gel at 100 V for 30 min. When the multi-band was formed, gel purification was performed, and when the single band was formed, PCR purification was performed. PCR and gel purification were conducted via Wizard® SV gel and PCR Clean-up System Kit (Promega, San Luis Obispo, CA, USA) according to the manufacturer’s instructions. The purified PCR product was sequenced in both directions via Bionics Co., Ltd. (Seoul, Korea) using EF1 and EF2 primer for TEF and 5f2, 7cr, 7cF, and 11aR primers for RPB2 (Supplementary Table S7). The consensus sequences were assembled and revised using the Seqman program (DNASTAR, Madison, USA)^37^. The novel sequences generated in this study were deposited in National Centre for Biotechnology Information (NCBI) GenBank.

### Phylogenetic analysis

To identify the species of the isolates, the sequence alignments of the TEF and RPB2 regions were conducted using the MUSCLE algorithm of MEGA-X software^38,39^ with other reference sequences of *Fusarium* spp. obtained from the NCBI GenBank. The phylogenetic trees were constructed based on the Maximum likelihood and Kimura 2-parameter model^40,41^ and verified by 1,000 bootstrap replicates^31^. The *F. staphyleae* strain NRRL 22316 was used as an out group. Information regarding the reference and outgroup strains is summarized in Supplementary Table S8.

### Pathogenicity test

The pathogenicity test of the 29 isolates was conducted via the soil inoculation method using cornmeal sand inoculum to the original host (host from which each isolate was collected)^42^. The cornmeal sand inoculum was prepared by mixing 240 g dry sand, 26 g cornmeal, and 65 ml distilled water in 500-mL Erlenmeyer flasks, autoclaving twice at 121 ℃ for 30 min, and adding 15 PDA disks (5-mm diameter) with pathogen mycelium. In the control treatment, pure PDA disks were added instead of inoculated disks. The inoculum was incubated at 25 ℃ for 4 weeks without shaking. Following inoculation, the cornmeal sand inoculum and autoclaved soil were mixed at a volume ratio of 3:7 and then divided into 200 mL for each pot (72 × 72 × 100 mm). Two germinated seeds were planted in each pot, and three replicates pots were used for each treatment. All plants were grown in the controlled plant growth room at 25 ℃–27 ℃, with a photoperiod of 12 h/day. Three weeks after sowing, the disease index was scored on a 0–4 scale for each host according to the degree of root damage (Supplementary Fig. S5).

### Investigation of host range

The host range investigation was conducted with selected isolates based on their pathogenicity; hence, certain isolates with low virulence were also included. In total, 19 isolates were investigated, including 6 isolates collected from kidney beans, 5 from mung beans, 5 from adzuki beans, and 3 from sword beans. The host range investigation assay was also conducted using the soil inoculation method with cornmeal sand inoculum, similar to the pathogenicity test. However, there were some differences in the experimental procedures. In this assay, on preparing the cornmeal sand inoculum, 450 mL dry sand, 26 g finely ground cornmeal (for food), and 70 mL distilled water were mixed in a 1-L Erlenmeyer flask, and 30 PDA disks (5-mm diameter) inoculated with pathogens were added. After incubation for 4 weeks at 25 ℃, the mixture of cornmeal sand inoculum and sterilized soil in a 2:8 volume ratio was placed in 100 × 40 mm plant culture dishes with holes in their bottom, and eight seeds were planted for each crop. The plants used for this host range assay included nine crop plants, involving seven leguminous plants and two gramineous plants. The leguminous crops included kidney bean, mung bean, lentil, sword bean, soybean, adzuki bean, and cowpea. Furthermore, the gramineous plants included rice and corn. The disease index was evaluated 3 weeks after inoculation according to the degree of root damage, similar to the pathogenicity test. Then, Duncan’s DMRT was performed at a 5% significance level using the R program to statistically confirm whether there was a significant difference in the incidence of strains for the host.

### Supplementary Information

Supplementary Information.

## Data Availability

All sequences produced in this study are publicly available in NCBI GenBank Database (https://www.ncbi.nlm.nih.gov/genbank/) and accession numbers are available in Supplementary Table S6. The datasets used and/or analyzed during the current study were available from the corresponding author on reasonable request.
