# Supplementary material for: Diversity and pathogenic characteristics of the Fusarium species isolated from minor legumes in Korea
Source: Sci Rep. 2023 Dec 18;13:22516. doi: 10.1038/s41598-023-49736-4 (PMC10728068; doi:10.1038/s41598-023-49736-4)
Supplement: Supplementary file 1 — Supplementary Information. [file 41598_2023_49736_MOESM1_ESM.docx]

**Supplementary information:**

**Diversity and pathogenic characteristics of *Fusarium* species isolated from minor legumes in Korea**

Min Sun Ha^1,3^, Hyunjoo Ryu^1^, Ho Jong Ju^3,4‡^, and Hyo-Won Choi^2†^*

^1^Crop Protection Division, National Institute of Agricultural Sciences, Wanju 55365, Korea.

^2^Extension Service Bureau Disaster Management division, Rural Development Administration, Jeonju, 54875, Korea.

^3^Department of Agricultural Biology, Jeonbuk National University, Jeonju 54896, Korea.

^4^Institute of Agricultural Science and Technology, Jeonbuk National University, Jeonju 54896, Korea.

^†^Corresponding author: [hyon338@korea.kr](mailto:hyon338@korea.kr), ^‡^Equally contributed corresponding author.

**Supplementary Figures**

**
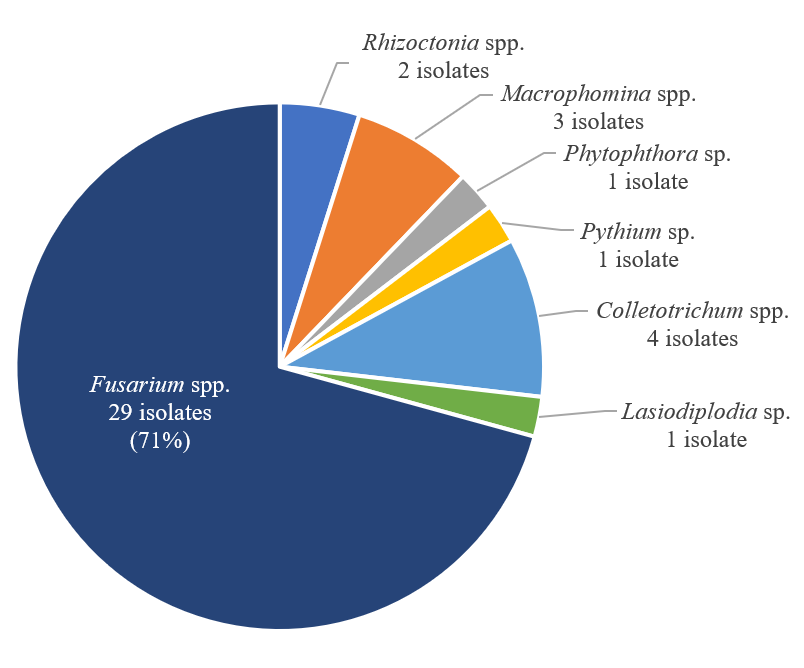
**

**Supplementary Figure S1.** Isolation rate of fungi obtained from legume plant exhibiting wilt symptoms. Among 41 isolates, *Fusarium* was the dominant genus, accounting for 71% (29) of the isolates.


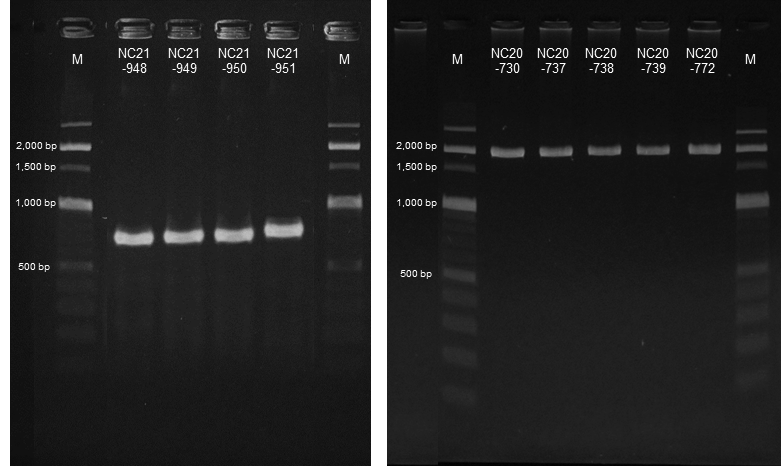


**Supplementary Figure S2. Agarose gel electrophoresis (1.4%) of PCR amplification products for target band sequencing of *Fusarium* spp. isolated from minor legumes.** The 600-800 bp of TEF (left) and 2,000 bp of RPB2 (right) regions were amplified. Lane M showing 100 bp DNA ladder.


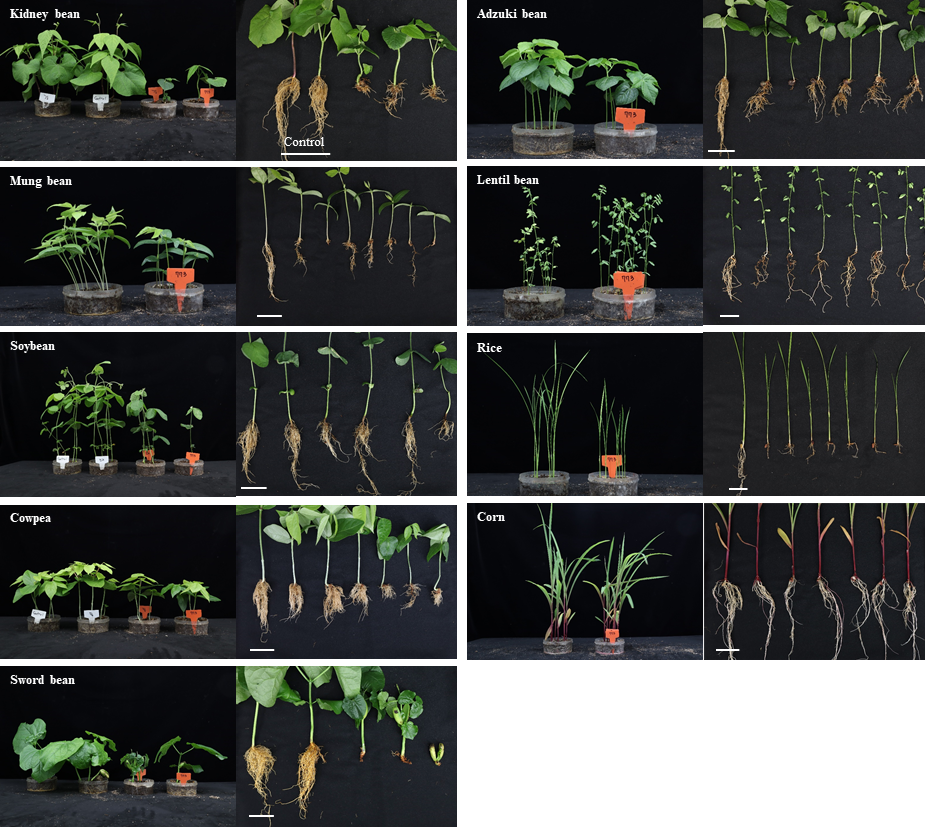


**Supplementary Figure S3. Host range of *Fusarium oxysporum* NC20-773.** These are pictures of the above-ground and underground parts of 7 leguminous and 2 gramineous crops 3 weeks after inoculation. The orange label in the above-ground are the plants inoculated with NC20-773, and the left one is the control plant. In the photos of the underground part, the white bar is the control plant, and all the rest are the plants inoculated with NC20-773.


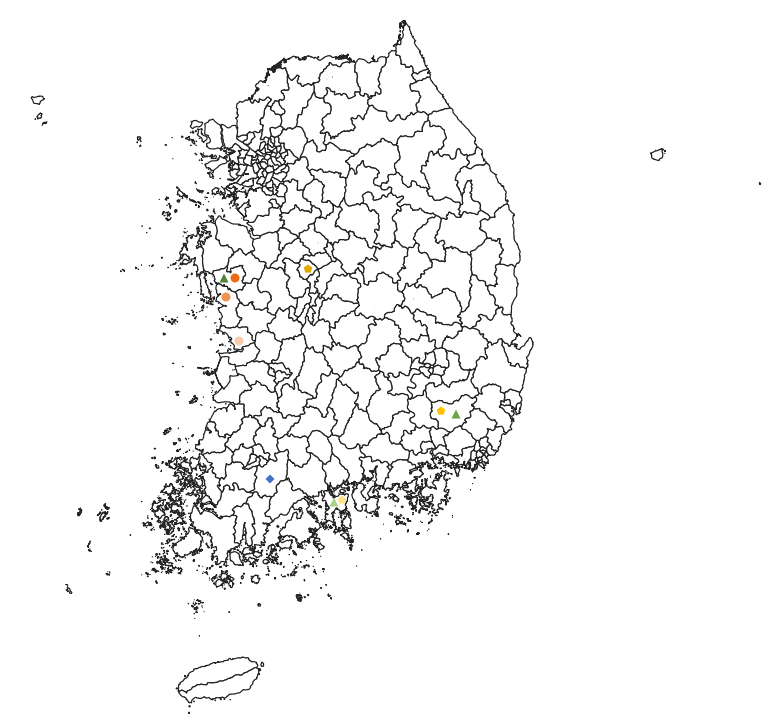

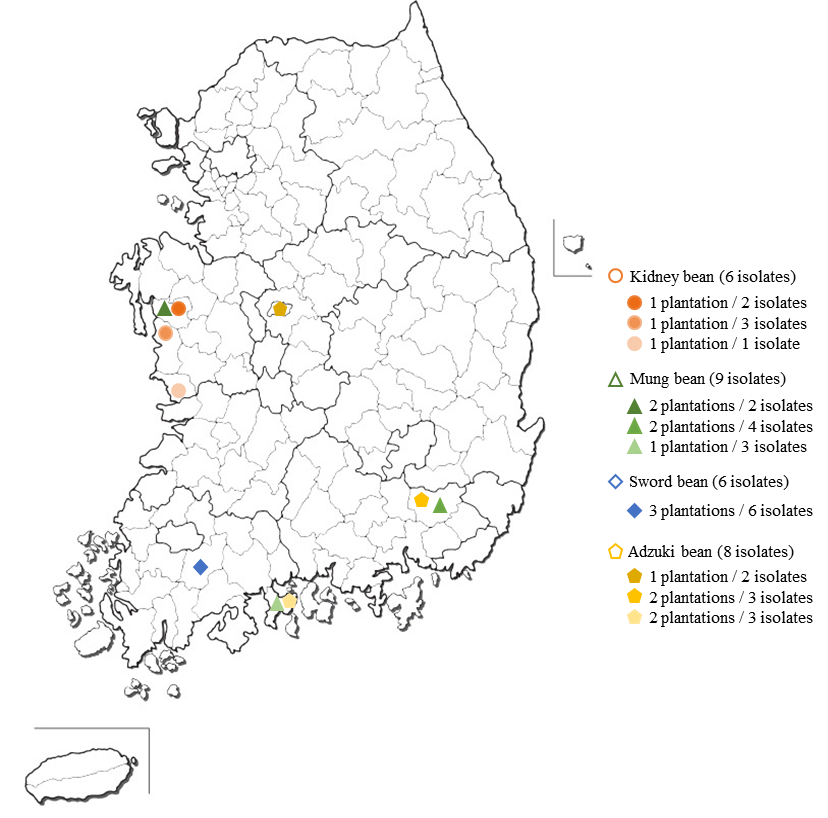


**Supplementary Figure S4. Map showing sampling locations and number of isolates.** From 2020 to 2021, samples exhibiting wilt symptoms from minor legumes were collected from 14 domestic legume plantations in Hwasun, Hongseong, Cheongju, Yeosu, Miryang, Boryeong, Seocheon. The map was created using QGIS software version 3.28 (https://www.qgis.org/).


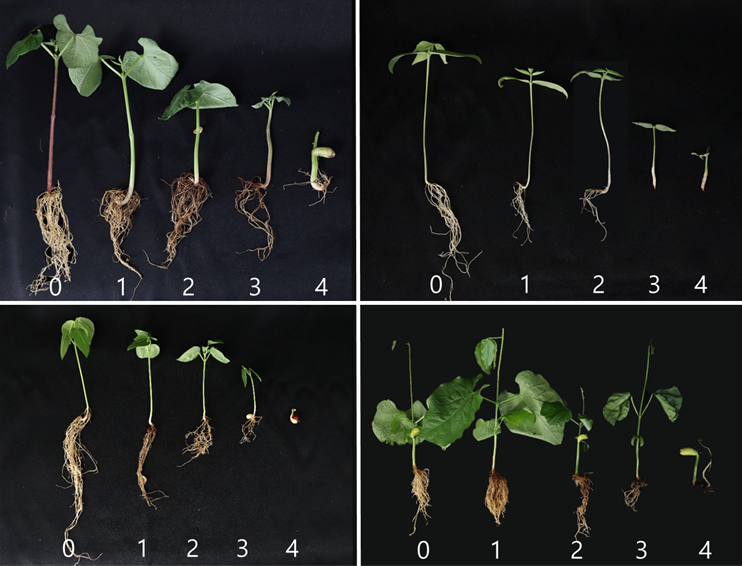


a

b

c

d

**Supplementary Figure S5.** Disease index of four leguminous crops, which are the original hosts of the isolates. The disease index of a) kidney beans, b) mung beans, c) adzuki beans, and d) sword beans. Disease index 0 = no symptoms, 1 = root necrosis and root loss <30%, 2 = root necrosis and root loss 31%–60%, 3 = root necrosis, root loss >61%, and poor growth, and 4 = complete necrosis of root tissue and no roots or plants death.

**Supplementary Tables**

**Supplementary Table S1. Morphological and cultural characteristics of the 13** **isolates belong to *F. solani* species complex (FSSC)**

| Structure^a^ | | Characteristics | | | | | | | | | | | | |
| --- | --- | --- | --- | --- | --- | --- | --- | --- | --- | --- | --- | --- | --- | --- |
|  |  | FSSC | | | | | | | | | | | | |
|  |  | NC20-728 | NC20-734 | NC20-729 | NC20-731 | NC20-774 | NC20-743 | NC21-951 | NC20-745 | NC20-776 | NC21-952 | NC21-953 | NC21-982 | NC21-983 |
|  |  | *F. vanettenii* | *F. vanettenii* | *F. azukicola* | *F. falciforme* | *F. falciforme* | *F. solani* | *F. solani* | *F. phaseoli* | *F. oblongum* | *F. ferrugineum* | *F. liriodendri* | *F. metavorans* | *F. metavorans* |
| Growth rate (mm/day) | | 9-10 | 9-10 | 3-4 | 10 | 9-10 | 10-11 | 9-10 | 3-4 | 9-10 | 10-11 | 10-11 | 9-10 | 9-10 |
| Aerial mycelium | | Sparse, white to beige | Sparse, white to beige, flat | Abundant, pink with white margin | Sparse, white to cream, felt-like | White, flat, felt-like | Sparse, white to beige, felt-like | Rather sparse, white to cream, felt-like | Abundant, grayish-white, white, wrinkle | Sparse, white to cream, felt-like | Sparse, white to dark magenta, felt-like | Rather sparse, white to grayish-red, felt-like | Sparse, white to dark magenta, felt-like | Sparse, white to cream, felt-like |
| Pigmentation | | Cream to buff | Cream to buff | Brownish orange with diffusing reddish brown | Cream to yellowish cream, light pink | Cream to buff, brown | Cream to buff, light pink | Cream to buff, light orange | Brownish orange | Cream to buff | Buff to brownish-orange | Buff to brownish-orange | Grayish-yellow to red | Cream to buff |
| Macroconidia | Shape  (entire shape/ apical cell/ basal cell) | Slightly curved / blunt / distinct foot-shaped | Slightly curved / blunt / distinct foot-shaped | Falcate / slightly rostrate / poorly developed foot-shaped, rounded | Rather thick-walled, slightly curved /blunt / papillate | Straight, slightly curved /blunt / papillate | Rather thick-walled, straight / blunt / poorly developed foot-shaped | Straight / blunt / papillate | Falcate, straight / blunt / rounded | Slightly curved / blunt / papillate, poorly developed foot-shaped | Slightly curved / blunt / poorly developed foot-shaped | Slightly curved / blunt / poorly developed foot-shaped | Straight / blunt / papillate | Straight / blunt / papillate |
|  | Septate | 3-5 | 3-5 | 3-4 | 3-4 | 3-5 | 3-4 | 3-5 | 2-4 | 3-5 | 5-6 | 3-6 | 5-7 | 5-7 |
|  | Size (µm) | 43-60×  5.4-6.6 | 46.7-56.2  ×4.5-6 | 72.9-83.6  ×4.5-5.4 | 38.6-52.4  ×4.6-6 | 41.9-50.8  ×5.2-7 | 30.8-44.9  ×3.4-5 | 39.3-48.2  ×5.4-7.2 | 40.8-52.9  ×5.1-7.2 | 42.1-55  ×5.9-2.7 | 53.1-66.7  ×4.8-5.9 | 46.7-72.6  ×4.1-6.5 | 52.5-64.9  ×5-6.3 | 52.5-64.9  ×5-6.3 |
| Microconidia | Shape | Oval, obovoid | Oval, obovoid | Absent | Obovoid, clavate | Oval, obovoid, clavate | Oval, obovoid | Oval, obovoid | Absent | Oval, obovoid | Oval, obovoid, clavate | Oval, obovoid, clavate | Oval, obovoid | Oval, obovoid |
|  | Septate | 0-1 | 0-1 |  | 0-1 | 0-1 | 0-1 | 0-1 |  | 0-1 | 0-1 | 0-1 | 0-1 | 0-1 |
|  | Size (µm) | 6.7-20  ×2.9-4.7 | 6.7-11.4  ×2.9-4.7 |  | 8.3-20.4  ×2.3-4.8 | 8.6-24  ×2.6-5 | 5.2-12  ×2-3.3 | 7.4-17.2  ×2.8-5.7 |  | 4.5-8.7  ×1.9-3.8 | 5.5-10.9  ×2.1-3.9 | 4.2-10.2  ×1.7-4.1 | 4.2-8  ×1.7-3.7 | 5-10  ×2-4.6 |
| Sporodochia | | Absent | Abundant, white to beige | White | Abundant, white to beige | Abundant, white to beige | Abundant, pale orange | White to beige | Beige | White to beige | Abundant, beige | Abundant, white to blue | Abundant, white to beige | Abundant, white to beige |

^a^ Growth rate, aerial mycelium and pigmentation were investigated on PDA plates incubated in darkness at 25˚C for a week. Other structures were investigated on CLA plates incubated in alternating cycles of 12h NUV light and 12h darkness at 25˚C for 2 weeks.

**Supplementary Table S2. Morphological and cultural characteristics of the 7 isolates belong to *F. oxysporum* species complex (FOSC)**

| Structure^a^ | | Characteristics | | | | | | |
| --- | --- | --- | --- | --- | --- | --- | --- | --- |
|  |  | FOSC | | | | | | |
|  |  | NC20-730 | NC20-733 | NC20-742 | NC20-746 | NC20-772 | NC20-773 | NC20-779 |
|  |  | *F. oxysporum* | *F. oxysporum* | *F. oxysporum* | *F. oxysporum* | *F. oxysporum* | *F. oxysporum* | *F. oxysporum* |
| Growth rate (mm/day) | | 11-12 | 10-11 | 11-12 | 12 | 11 | 9-10 | 11-12 |
| Aerial mycelium | | Sparse, white | Sparse, white | Abundant, white, floccose | Abundant, white, floccose | Sparse, white, felt-like | Abundant, white, floccose | Abundant, white to pale violet, floccose |
| Pigmentation | | Absent | Grayish-violet | Buff to violet | Bright pink | Cream to buff | Cream to pale violet | Dark violet |
| Macroconidia | Shape  (entire shape/ apical cell/ basal cell) | Straight, moderately curved / blunt, slightly hooked / papillate, poorly developed foot-shaped | Slightly curved / pointed, slightly hooked / poorly developed foot-shaped | Straight / blunt / obtuse, distinct foot-shaped | Straight / blunt / distinct foot-shaped | Slightly curved / blunt /obtuse, papillate | Straight / pointed, blunt / distinct foot-shaped | Moderately curved / pointed / distinct foot-shaped |
|  | Septate | 3-4 | 3-4 | 3-4 | 3-4 | 3-4 | 3-4 | 3-4 |
|  | Size (µm) | 28.5-44.6 ×3.5-5.6 | 37.3-48.8 ×3.8-5.1 | 25.2-38.2 ×3.6-4.2 | 27.1-43.4 ×3.3-5.2 | 34.9-44.8 ×4.6-6.2 | 31.4-42 ×3.1-5.2 | 41.1-51.5 ×3.5-5.1 |
| Microconidia | Shape | Cylindric, oval, obovoid, reniform | Oval, reniform | Cylindric, obovoid, reniform | Cylindric, oval, allantoid | Cylindric, oval | Cylindric, obovoid | Cylindric, oval |
|  | Septate | 0-1 | 0-1 | 0-1 | 0-1 | 0-1 | 0-1 | 0-1 |
|  | Size (µm) | 5-14.8×2.4-4.2 | 7.1-15.9 ×3.4-4.6 | 7.3-12.1 ×2.1-3.5 | 6.6-17.8 ×2.1-17.5 | 7.7-12.8×2.9-4 | 5.8-11×2.2-4.1 | 7.6-12.8 ×2.4-3.8 |
| Sporodochia | | Absent | Absent | Absent | Absent | Abundant, white | Beige to pale orange | Abundant, orange |

^a^ Growth rate, aerial mycelium and pigmentation were investigated on PDA plates incubated in darkness at 25˚C for a week. Other structures were investigated on CLA plates incubated in alternating cycles of 12h NUV light and 12h darkness at 25˚C for 2 weeks.

**Supplementary Table S3. Morphological and cultural characteristics of the 7 isolates belong to *F. fujikuroi* species complex (FFSC)**

| Structure^a^ | | Characteristics | | | | | | |
| --- | --- | --- | --- | --- | --- | --- | --- | --- |
|  |  | FFSC | | | | | | |
|  |  | NC20-732 | NC20-737 | NC20-738 | NC20-739 | NC21-948 | NC21-949 | NC21-950 |
|  |  | *F. fujikuroi* | *F. fujikuroi* | *F. fujikuroi* | *F. fujikuroi* | *F. concentricum* | *F. proliferatum* | *F. proliferatum* |
| Growth rate (mm/day) | | 10-11 | 7-8 | 9-10 | 8-9 | 10-11 | 10-11 | 9-10 |
| Aerial mycelium | | Abundant, cottony, white to dark magenta | Abundant, cottony, white | Abundant, cottony, white | Abundant, cottony, whitish to pale violet | Abundant, cottony, white, irregular form | Abundant, cottony, white | Abundant, cottony, white |
| Pigmentation | | Rust brick (almost black) | Buff to rust brick | Pale violet to rust brick | Buff to rust brick | Cream | Cream | Buff |
| Macroconidia | Shape  (entire shape/ apical cell/ basal cell) | Slender, straight / curved / distinct foot-shaped | Slender, straight / blunt / distinct foot-shaped | Straight / blunt / distinct foot-shaped | Slender, straight / curved /distinct foot-shaped | Slender, straight / curved /distinct foot-shaped | Slender, straight / curved /distinct foot-shaped | Slender, straight / curved /distinct foot-shaped |
|  | Septate | 3-4 | 3-5 | 3-4 | 3-4 | 3-4 | 3-4 | 3-5 |
|  | Size (µm) | 34.7-53.1 ×3.2-4.3 | 29.2-54.7 ×2.6-4.5 | 32.5-45.6 ×3.5-5.1 | 33.2-53.3 ×2.2-4.6 | 38.1-58.4×3-4.5 | 41.8-55.4 ×3.3-4.1 | 34.6-54.7 ×2.6-3.6 |
| Microconidia | Shape | Clavate, obovoid | Clavate, obovoid | Clavate, oval | Clavate | Fusiform, oval, obovoid | Clavate, obovoid | Clavate, obovoid, cylindric |
|  | Septate | 0-1 | 0-1 | 0-1 | 0-3 | 0-1 | 0-1 | 0-1 |
|  | Size (µm) | 5.1-12.9×2.1-4.3 | 5.3-8.6×1.9-3.8 | 5.6-10.9×2-3.8 | 4.6-13.7×2-4.5 | 4.9-18.3×1.9-3.6 | 6.2-10.8×2.5-3.7 | 6.2-15.2×2-3.7 |
| Sporodochia | | Absent | Abundant, pale orange | Abundant, pale orange | Pale orange | Abundant, pale orange | Abundant, pale yellow | Absent |

^a^ Growth rate, aerial mycelium and pigmentation were investigated on PDA plates incubated in darkness at 25˚C for a week. Other structures were investigated on CLA plates incubated in alternating cycles of 12h NUV light and 12h darkness at 25˚C for 2 weeks.

**Supplementary Table S4. Morphological and cultural characteristics of the 2 isolates belong to *F. incarnatum-equiseti* species complex (FIESC)**

| Structure^a^ | | Characteristics | |
| --- | --- | --- | --- |
|  |  | FIESC | |
|  |  | NC20-777 | FD00137 |
|  |  | *F. ipomoeae* | *F. ipomoeae* |
| Growth rate (mm/day) | | 5-6 | 5 |
| Aerial mycelium | | Dense, chartreuse center, white margin | Dense, chartreuse center, white margin |
| Pigmentation | | Brownish orange to light pink | Reddish orange |
| Macroconidia | Shape  (entire shape / apical cell / basal cell) | Elongate, whip-like / hooked / elongated foot-shaped | Elongate, whip-like / hooked / elongated foot-shaped |
|  | Septate | 3-5 | 3-5 |
|  | Size (µm) | 62.6-75×2.6-4 | 51.4-65×3.2-4.8 |
| Microconidia | Shape | Absent | Absent |
|  | Septate |  |  |
|  | Size (µm) |  |  |
| Sporodochia | | Abundant, Pale orange | Abundant, Pale orange |

^a^ Growth rate, aerial mycelium and pigmentation were investigated on PDA plates incubated in darkness at 25˚C for a week. Other structures were investigated on CLA plates incubated in alternating cycles of 12h NUV light and 12h darkness at 25˚C for 2 weeks.

**Supplementary Table S5. Previously described morphological and cultural characteristics of 14 *Fusarium* species**

| Structure^a^ | | Characteristics^b^ | | | | | | | | | | | | | |
| --- | --- | --- | --- | --- | --- | --- | --- | --- | --- | --- | --- | --- | --- | --- | --- |
|  |  | *F. vanettenii* | *F. azukicola* | *F. falciforme* | *F. solani* | *F. phaseoli* | *F. oblongum* | *F. ferrugineum* | *F. liriodendri* | *F. metavorans* | *F. oxysporum* | *F. fujikuroi* | *F. concentricum* | *F. proliferatum* | *F. ipomoeae* |
| Growth rate (mm/day) | | 6-7 | 1-2 | 10-11 | Fast, 9-10 | 1-2 | 2-3 | 4-5 | 6-7 | 5-6 | Fast, 7-11 | Fast, 10-12 | 4-5 | 9-10 | 7-9 |
| Aerial mycelium | | Dense, cottony, white | Loose to floccose, white to reddish white, pale red | White, flat | Usually rather sparse, felt-like | Abundant, floccose, white to pastel gray | white, flat, velvety | rust to brick, flat, velvety | white, velvety to cottony | white to reddish-white | Abundant, delicate, floccose | Abundant, cottony, whitish to pale pink | Reddish-white, velvety to lanose | Abundant, white to pinkish buff | Dense, chartreuse center, pinkish-white margin |
| Pigmentation | | Yellowish to orange-brown | Absent or grayish yellow, grayish orange to brownish orange | Creamy to yellowish | Rather variable, cream to buff | Grayish-yellow to brownish-orange | Saffron to rosy buff | Apricot to rust | Buff to honey | Pale yellow (margin), dark brown to red (center) | Very variable (pale beige to violet) | Buff to rust brick | Pale orange to reddish-grey | Buff, grey-lilac, brick | Greyish orange center, pinkish white margin |
| Macroconidia | Shape  (entire shape/ apical cell/ basal cell) | Slightly curved/ round / pedicellate | Falcate, cylindric / slightly rostrate, rounded/ distinct foot-shaped, rounded | Straight to slightly curved / papillate / barely notched | Rather thick-walled, subcylindric / blunt / indistinctly pedicellate | Falcate / acuate /rounded but protruding | Absent | Moderately curved / blunt / distictly notched | Absent | Absent | Moderately curved / pointed, slightly hooked / distinctly pedicellate | Slender, straight / curved /distinct foot-shaped | Slender / slightly beaked / foot-shaped | Subfalcate / curved / distinctly foot-shaped | Dorsoventral curvature / hooked to tapering / foot-shaped |
|  | Septate | 2-4 | 3-5 | 3-5 | 3-5 | 2-5 |  | 4-7 |  |  | 3-5 | 3-5 | 3-5 | 3-5 | 3-5 |
|  | Size (µm) | 4.3-46.1  ×5.4-6.2 | 44-106  ×4.5-7 | 21.3-66.1  ×3.8-7.3 | 22-62  ×3.5-7 | 32.5-73.5  ×4-6.5 |  | 54-71.5  ×5-6.5 |  |  | 27-42  ×3-4.7 | 22-65  ×2.5-4.7 | 42-64.8  ×3.5-4 | 19-79  ×2.6-5 | 26.5-57  ×3-5 |
| Microconidia | Shape | Ovoidal | Ellipsoid to clavate, reniform | Oval | Oval, ellipsoid to subcylindric | Clavate to ellipsoid | Clavate, ellipsoid | Ellipsoid to allantoid | Obovoid, subcylindric to clavate | Ellipsoid | Cylindric to ellipsoid, oval, reniform | Clavate with a flattened base | Obovoid, oval to allantoid | Clavate, pyriform | Absent |
|  | Septate | 0-1 | 0-1 | 0-2 | 0-1 | 0-1 | 0-1 | 0-2 | 0-1 | 0-1 | 0-1 | 0-1 | 0-1 | 0-1 |  |
|  | Size (µm) | 13.5-32.5  ×3.5-6 | 4-13.5  ×2-4 | 3.9-25.7  ×2.2-5.1 | 5.0-27  ×2.8-7 | 4-13.5  ×2-4 | 5-22  ×2-5.5 | 4-20  ×1.5-6 | 4.5-16  ×2-5 | 3.9-11.1  ×2.3-3.1 | 5-9  ×2.4-3 | 5-13  ×2.1-4.7 | 7-12.2  ×2.3-3.9 | 3-16  ×1.2-12.4 |  |
| Sporodochia | | White | Abundant (sometimes sparse) | White to cream | White to beige | Abundant | Absent | White to buff | Absent | Absent | Pale salmon to bright orange | Abundant | Pale orange | Rare | Pale orange |

^a^ Growth rate, aerial mycelium and pigmentation were investigated on PDA plates incubated in darkness at 25˚C for a week. Other structures were investigated on CLA plates incubated in alternating cycles of 12h NUV light and 12h darkness at 25˚C for 2 weeks.

^b^ *F. solani, F. oxysporum, F. fujikuroi, F. proliferatum* described by Gerlach, et al. ^1^; *F. oblongum, F. ferrugineum, F. liriodendri* described by Sandoval-Denis, et al. ^2^; *F. ipomoeae*. described by Wang, et al. ^3^; *F. vanettenii* described by Sisic, et al. ^4^; *F.phaseoli* described by Aoki, et al. ^5^; *F. azukicola* described by Aoki, et al. ^6^; *F. falciforme* described by Balasubramaniam, et al. ^7^; *F. metavorans* described by Al-Hatmi, et al. ^8^; and *F. concentricum* described by Nirenberg and O'Donnell ^9^.

**Supplementary Table S6. GenBank accession numbers and information for the 29 isolates of *Fusarium* species used in this study**

| No. | Species complex^a^ | Species | Isolate | Host | Location | Accession numbers | |
| --- | --- | --- | --- | --- | --- | --- | --- |
|  |  |  |  |  |  | TEF | RPB2 |
| 1 | FSSC | *F. vanettenii* | NC20-728 | *Phaseolus vulgaris* | Boryeong, Chungnam | OP784429 | OP784439 |
| 2 |  | *F. azukicola* | NC20-729 | *Vigna radiata* | Yeosu, Jeonnam | ON548180 | ON638991 |
| 3 |  | *F. falciforme* | NC20-731 | *V. radiata* | Yeosu, Jeonnam | OP920659 | OP913396 |
| 4 |  | *F. vanettenii* | NC20-734 | *P. vulgaris* | Boryeong, Chungnam | OP784432 | OP784442 |
| 5 |  | *F. solani* | NC20-743 | *V. radiata* | Hongseong, Chungnam | OP920661 | OP913398 |
| 6 |  | *F. phaseoli* | NC20-745 | *P. vulgaris* | Hongseong, Chungnam | OP784433 | OP784443 |
| 7 |  | *F. falciforme* | NC20-774 | *V. angularis* | Miryang, Gyeongnam | OP920668 | OP913405 |
| 8 |  | *F. oblongum* | NC20-776 | *V. radiata* | Miryang, Gyeongnam | OP920663 | OP913400 |
| 9 |  | *F. solani* | NC21-951 | *Canavalia ensiformis* | Hwasun, Jeonnam | OP784437 | OP784447 |
| 10 |  | *F. ferrugineum* | NC21-952 | *C. ensiformis* | Hwasun, Jeonnam | OP784438 | OP784448 |
| 11 |  | *F. liriodendri* | NC21-953 | *C. ensiformis* | Hwasun, Jeonnam | OP920672 | OP920674 |
| 12 |  | *F. metavorans* | NC21-982 | *V. angularis* | Cheongju, Chungbuk | OP920669 | OP913406 |
| 13 |  | *F. metavorans* | NC21-983 | *V. angularis* | Cheongju, Chungbuk | OP920670 | OP913407 |
| 14 | FOSC | *F. oxysporum* | NC20-730 | *V. radiata* | Yeosu, Jeonnam | OP920658 | OP913395 |
| 15 |  | *F. oxysporum* | NC20-733 | *P. vulgaris* | Boryeong, Chungnam | OP784431 | OP784441 |
| 16 |  | *F. oxysporum* | NC20-742 | *V. radiata* | Hongseong, Chungnam | OP920660 | OP913397 |
| 17 |  | *F. oxysporum* | NC20-746 | *P. vulgaris* | Hongseong, Chungnam | OP784434 | OP784444 |
| 18 |  | *F. oxysporum* | NC20-772 | *V. angularis* | Miryang, Gyeongnam | OP920666 | OP913403 |
| 19 |  | *F. oxysporum* | NC20-773 | *V. angularis* | Miryang, Gyeongnam | OP920667 | OP913404 |
| 20 |  | *F. oxysporum* | NC20-779 | *V.radiata* | Miryang, Gyeongnam | OP920665 | OP913402 |
| 21 | FFSC | *F. fujikuroi* | NC20-732 | *P. vulgaris* | Miryang, Gyeongnam | OP784430 | OP784440 |
| 22 |  | *F. fujikuroi* | NC20-737 | *V. angularis* | Miryang, Gyeongnam | ON548181 | ON638992 |
| 23 |  | *F. fujikuroi* | NC20-738 | *V. angularis* | Hwasun, Jeonnam | ON548182 | ON638993 |
| 24 |  | *F. fujikuroi* | NC20-739 | *V. angularis* | Hwasun, Jeonnam | ON548183 | ON638994 |
| 25 |  | *F. concentricum* | NC21-948 | *C. ensiformis* | Hwasun, Jeonnam | OP784435 | OP784445 |
| 26 |  | *F. proliferatum* | NC21-949 | *C. ensiformis* | Cheongju, Chungbuk | OP784436 | OP784446 |
| 27 |  | *F. proliferatum* | NC21-950 | *C. ensiformis* | Cheongju, Chungbuk | OP920671 | OP920673 |
| 28 | FIESC | *F. ipomoeae* | NC20-777 | *V. radiata* | Miryang, Gyeongnam | OP920664 | OP913401 |
| 29 |  | *F. ipomoeae* | FD00137 | *V. radiata* | Miryang, Gyeongnam | OP957422 | OP940096 |

^a^ FSSC, *F. solani* species complex; FOSC, *F. oxysporum* species complex; FFSC, *F. fujikuroi* species complex; FIESC, *F. incarnatum-equiseti* species complex.

**Supplementary Table S7. Primer list used for PCR and sequencing**

| Locus | Gene product | Amplification size (bp) | Designation | Sequence^a^ (5’-3’) | Usage^b^ | | References |
| --- | --- | --- | --- | --- | --- | --- | --- |
|  |  |  |  |  | PCR | Sequencing |  |
| TEF | Translation elongation  factor 1α | 600-800 | EF1 | ATGGGTAAGGARGACAAGAC | ● | ● | O’Donnell, et al. ^10^ |
|  |  |  | EF2 | GGARGTACCAGTSATCATG | ● | ● | O’Donnell, et al. ^10^ |
| RPB2 | RNA polymerase  second largest subunit | 1,800-2,000 | 5f2 | GGGGWGAYCAGAAGAAGGC | ● | ● | Reeb, et al. ^11^ |
|  |  |  | 7cr | CCCATRGCTTGYTTRCCCAT |  | ● | Liu, et al. ^12^ |
|  |  |  | 7cF | ATGGGYAARCAAGCYATGGG |  | ● | Liu, et al. ^12^ |
|  |  |  | 11aR | GCRTGGATCTTRTCRTCSACC | ● | ● | Liu, et al. ^12^ |

^a^ D = A, G or T; R = A or G; S = C or G; W = A or T; Y = C or T.

^b^ ●, Primer was used for indicated purpose.

C

**Supplementary Table S8. List of reference *Fusarium* species used for phylogenetic analysis in this study**

| Species complex^a^ | Species | Strain | Host | Location | Accession numbers | |
| --- | --- | --- | --- | --- | --- | --- |
|  |  |  |  |  | TEF | RPB2 |
| FSSC | *F. ferrugineum* | NRRL 32437 | Human | Switzerland | DQ246979 | EU329581 |
|  | *F. liriodendri* | NRRL 22389 | *Liriodendron tulipifera* | USA | AF178340 | EU329506 |
|  | *F. oblongum* | NRRL 28008 | Human | USA | DQ246868 | - |
|  |  | LC7499 | Carbonatite | China | - | MW474704 |
|  | *F. vanettenii* | NRRL 22278 | *Pisum sativum* | USA | AF178337 | EU329501 |
|  |  | NRRL 22820 | *Glycine max* | USA | AF178355 | EU329532 |
|  | *F. metavorans* | NRRL 43489 | Human | USA | DQ790484 | DQ790572 |
|  |  | NRRL 22792 | Human | Spain | DQ246854 | EU329531 |
|  | *F. solani* | GJS 09–1466 | *Solanum tuberosum* | Slovenia | KT313611 | KT313623 |
|  |  | LC5548 | Soil | China | MW620200 | MW474725 |
|  | *F. falciforme* | NRRL 32757 | Sand | USA | DQ247075 | EU329614 |
|  |  | NRRL 43441 | Human | USA | MH582417 | MH582407 |
|  | *F. azukicola* | NRRL 54366 | *Vigna angularis* | Japan | JQ670139 | KJ511288 |
|  | *F. phaseoli* | NRRL31156 | Soil | USA | AY220187 | FJ240388 |
| FOSC | *F. oxysporum* | BRIP29093 | *Musa* sp. | Australia | KX434886 | KX434956 |
|  | *F. oxysporum f. sp. cubense* | NRRL25609 | *Musa* sp. | Malawi | KX434895 | KX434965 |
|  |  | NRRL26029 | *Musa* sp. | USA | KX434896 | KX434966 |
|  | *F. foetens* | CBS 110286 | Sand | USA | MT011001 | MW928825 |
|  | *F. inflexum* | NRRL 20433 | *Vicia faba* | Germany | AF008479 | JX171583 |
| FFSC | *F. fujikuroi* | NRRL 5538 | *Saccharum officinarum* | Taiwan | MN193860 | MN193888 |
|  |  | NRRL 13566 | *Oryza sativa* | Taiwan | AF160279 | JX171570 |
|  | *F. proliferatum* | NRRL 32155 | *Cicer arietinum* | India | FJ538242 | - |
|  |  | NRRL 62905 | *Zea mays* | USA | - | MN193893 |
|  |  | NRRL 25028 | *Melanaspis glomerata* | India | JF740705 | JF741035 |
|  | *F. concentricum* | CBS 450.97 | *Musa sapientum* | Costa Rica | MT010992 | MT010981 |
|  |  | CBS 453.97 | *M. sapientum* | Guatemala | MN533998 | MN534264 |
| FIESC | *F. ipomoeae* | LC6926 | *O. sativa* | China | MK289619 | MK289773 |
|  |  | LC12165 | *Ipomoea aquatica* | China | MK289599 | MK289752 |
|  | *F. equiseti* | NRRL 20697 | *Beta vulgaris* | Chile | GQ505594 | GQ505772 |
|  | *F. citri* | LC6896 | *Citrus reticulata* | China | MK289617 | MK289771 |
|  | *F. compactum* | NRRL 36323 | Cotton yarn | UK | GQ505648 | GQ505826 |
|  | *F. flagelliforme* | NRRL 36269 | *Pinus nigra* | Croatia | GQ505645 | GQ505823 |
| Outgroup | *F. staphyleae* | NRRL 22316 | *Staphylea trifolia* | USA | AF178361 | JX171609 |

^a^ FSSC, *F. solani* species complex; FOSC, *F. oxysporum* species complex; FFSC, *F. fujikuroi* species complex; FIESC, *F. incarnatum-equiseti* species complex.

**References**

1 Gerlach, W., Nirenberg, H., Eckart, I., Rummland, I. & Schwarz, R. *The genus Fusarium: a pictorial atlas*. Vol. 209 9-386 (Kommissionsverlag P. Parey Berlin, 1982).

2 Sandoval-Denis, M., Lombard, L. & Crous, P. Back to the roots: a reappraisal of Neocosmospora. *Persoonia-Molecular Phylogeny and Evolution of Fungi* **43**, 90-185 (2019).

3 Wang, M. M., Chen, Q., Diao, Y. Z., Duan, W. J. & Cai, L. *Fusarium incarnatum*-*equiseti* complex from China. *Persoonia* **43**, 70-89 (2019). <https://doi.org:10.3767/persoonia.2019.43.03>

4 Sisic, A. *et al.* The 'forma specialis' issue in *Fusarium*: A case study in *Fusarium solani* f. sp. *pisi*. *Sci Rep* **8**, 1252 (2018). <https://doi.org:10.1038/s41598-018-19779-z>

5 Aoki, T., O'Donnell, K., Homma, Y. & Lattanzi, A. R. Sudden-death syndrome of soybean is caused by two morphologically and phylogenetically distinct species within the *Fusarium solani* species complex—*F. virguliforme* in North America and *F. tucumaniae* in South America. *Mycologia* **95**, 660-684 (2003).

6 Aoki, T. *et al.* *Fusarium azukicola* sp. nov., an exotic azuki bean root-rot pathogen in Hokkaido, Japan. *Mycologia* **104**, 1068-1084 (2012). <https://doi.org:10.3852/11-303>

7 Balasubramaniam, J. *et al.* *Fusarium falciforme* and *F. oxysporum* causing postharvest fruit rot of watermelon (Citrullus lanatus) in Malaysia: A first report. *Crop Protection* **163**, 106115 (2023).

8 Al-Hatmi, A. M. *et al.* *Fusarium metavorans* sp. nov.: the frequent opportunist ‘FSSC6’. *Medical Mycology* **56**, S144-S152 (2018).

9 Nirenberg, H. I. & O'Donnell, K. New *Fusarium* species and combinations within the *Gibberella fujikuroi* species complex. *Mycologia* **90**, 434-458 (1998).

10 O’Donnell, K., Kistler, H. C., Cigelnik, E. & Ploetz, R. C. Multiple evolutionary origins of the fungus causing Panama disease of banana: concordant evidence from nuclear and mitochondrial gene genealogies. *Proceedings of the National Academy of Sciences* **95**, 2044-2049 (1998).

11 Reeb, V., Lutzoni, F. & Roux, C. Contribution of RPB2 to multilocus phylogenetic studies of the euascomycetes (Pezizomycotina, Fungi) with special emphasis on the lichen-forming Acarosporaceae and evolution of polyspory. *Molecular phylogenetics and evolution* **32**, 1036-1060 (2004).

12 Liu, Y. J., Whelen, S. & Hall, B. D. Phylogenetic relationships among ascomycetes: evidence from an RNA polymerse II subunit. *Molecular biology and evolution* **16**, 1799-1808 (1999).
